# Problem Drinking Predicts Functional Health and Mortality Risk 10 Years Later in the MIDUS Study

**DOI:** 10.1093/geroni/igaa057.1300

**Published:** 2020-12-16

**Authors:** Sara Miller, David Almeida, Jennifer Maggs

**Affiliations:** Pennsylvania State University, University Park, Pennsylvania, United States

## Abstract

The current study examined whether problem drinking in older adulthood is associated with greater longitudinal risk of functional impairment and mortality through 2016. Problem drinking consists of patterns of alcohol use resulting in symptoms of alcohol dependence or health and social consequences. Participants were adults (n=2654, 56.1% female) from Wave 2 (mean age=55, range=30-84) and Wave 3 (mean age=64, range=39-93) of the Survey of Midlife Development in the United States (MIDUS) Study. Participants reported problem drinking behaviors (e.g., alcohol related role interference) and any disability in basic and instrumental activities of daily living (ADL, iADL). Mortality data was acquired from the 2016 MIDUS Mortality dataset. Results indicated that 20.7% of the sample reported at least one problem drinking behavior in the past year. Multiple linear regression analyses revealed that the sum of problematic drinking behaviors at Wave 2 predicted 10-year longitudinal change in impairments in ADL’s (b=0.05, p<0.01) and iADL’s (b=0.05, p<0.01) after controlling for age, education, gender, and previous ADL/iADL. Logistic regression results revealed that for every additional alcohol use problem reported at MIDUS 2, the odds of mortality increased by 1.74 (b=0.55, p<0.01), beyond controls for age and number of chronic conditions. The findings that problem drinking has a unique positive association with impaired functioning and mortality risk during older adulthood supports public health efforts to encourage reduced consumption, increased medical screening, and expanded treatment options.

